# The serum protein responses to treatment with Xiaoke Pill and Glibenclamide in type 2 diabetes patients

**DOI:** 10.1186/s12014-017-9154-0

**Published:** 2017-05-17

**Authors:** Xiuying Zhang, Haidan Sun, Sanjoy K. Paul, Quanhui Wang, Xiaomin Lou, Guixue Hou, Bo Wen, Linong Ji, Siqi Liu

**Affiliations:** 1 Department of Endocrinology and Metabolism, Peking University People’s Hospital, Peking University Diabetes Centre, No. 11, Xi Zhi Men Nan Da Jie, Xicheng District, Beijing, 100044 China; 2 CAS Key Laboratory of Genome Sciences and Information, Beijing Institute of Genomics, Chinese Academy of Sciences, Beijing, 100101 China; 3 Clinical Trials and Biostatistics Unit, QIMR Berghofer Medical Research Institute, Brisbane, Australia; 4 Proteomics Division, BGI-Shenzhen, Shenzhen, 518083 China; 5 Institute of Basic Medical Sciences, Chinese Academy of Medical Sciences, School of Basic Medicine, Peking Union Medical College, Beijing, 100005 China

**Keywords:** Type 2 diabetes mellitus, Hypoglycemia, Proteomics, Glibenclamide, Chinese herb

## Abstract

**Aim:**

The Xiaoke Pill containing Chinese herb extracts and Glibenclamide, is used in therapy for type 2 diabetes mellitus (T2DM), and is effective in reducing the risk of hypoglycemia and improving diabetes symptoms compared with Glibenclamide. We describe a quantitative proteomics project to measure the T2DM serum proteome response to the Xiaoke Pill and Glibenclamide.

**Methods:**

Based on a recently conducted 48-week clinical trial comparing the safety and efficacy of Glibenclamide (n = 400) and Xiaoke Pill (n = 400), after matching for age, sex, BMI, drug dose and whether hypoglycemia occurred, 32 patients were selected for the serum based proteomic analysis and divided into four groups (with/without hypoglycemia treated with Xiaoke Pill or Glibenclamide, n = 8 for each group). We screened the differential serum proteins related to treatments and the onset of hypoglycemia using the iTRAQ labeling quantitative proteomics technique. Baseline and follow-up samples were used.

**Results:**

The quantitative proteomics experiments demonstrated that 25 and 21 proteins differed upon treatment with the Xiaoke Pill in patients without and with hypoglycemia, respectively, while 24 and 25 proteins differed upon treatment with Glibenclamide in patients without and with hypoglycemia, respectively. The overlap of different proteins between the patients with and without hypoglycemia given the same drug treatment was much greater than between the patients given different drug treatments.

**Conclusions:**

We conclude that the serum proteins response to the two different anti-diabetic drug treatments may serve as a sensitive biomarker for evaluation of the therapeutic effects and continue investigations into the mechanism.

**Electronic supplementary material:**

The online version of this article (doi:10.1186/s12014-017-9154-0) contains supplementary material, which is available to authorized users.

## Background

Type 2 diabetes mellitus (T2DM) results from multiple genetic and environmental interactions. A primary side-effect of widely used anti-diabetic drugs, such as Glibenclamide, is hypoglycemia, which increases the risk of cardiovascular events [1]. Traditional Chinese medicine (TCM) exhibits therapeutic effects in many diseases, including as adjuvants to routine anti-diabetic drugs in anti-diabetic therapy [2, 3]. The Xiaoke Pill containing components from Chinese herb extracts and Glibenclamide, is an approved medicine in China that is often used to treat sweet urine, dry mouth, thirst, polydipsia, polyorexia, polyphagia, emaciation, and fatigue [4]. This medicine was also used in T2DM treatments and improves insulin secretion [5]. Recently, we investigated the safety and efficacy of treatment with Xiaoke Pill and Glibenclamide in patients with T2DM, wherein 800 patients were randomly assigned into two groups, Xiaoke Pill or Glibenclamide [6]. Patients in the Xiaoke Pill group significantly reduced the risk of hypoglycemia compared to those in the Glibenclamide group. Our observations demonstrate that the Xiaoke Pill may be advantageous in treating diabetes, especially for reducing the risk of hypoglycemia and improving diabetes symptoms. However, the basis for this result remains unclear.

Quantitative proteomics is a useful technique for estimating of the overall serum protein changes in response to stimuli. The approach using isobaric tags for relative and absolute quantitation (iTRAQ) is generally accepted in quantitative proteomics using multiple groups because the MS/MS intensities of the tags offer high quality signals for quantification, while the shared peptides enhance the detection sensitivity and allow quantification within eight parallel groups [7, 8]. Based on the approach of iTRAQ, the protective effects of TCM phlorizin in diabetic cardiomyopathy using db/db mice were estimated, and the differential urinary proteins in microalbuminuric versus normoalbuminuric type 2 diabetic patients were screened [9, 10]. Thus, a quantitative analysis using iTRAQ may provide solid data such that integrating the serum protein changes and clinical information derived from the Xiaoke Pill treatment will likely aid in controlling hypoglycemia through anti-diabetic drugs.

## Methods

### Subjects

The study design, study participants and the results of the Xiaoke Pill trial have already been reported [6]. Briefly, it was a controlled, double blind, multicentre non-inferiority trial, where 800 patients with unsatisfactory glycemic control were randomly assigned to receive Xiaoke Pill, a compound of Chinese herbs combined with Glibenclamide, or Glibenclamide under two study groups—drug naive group, and patients previously treated with metformin monotherapy. Outcome measures at 48 weeks were the incidence and rate of hypoglycemia, change in glycated hemoglobin (HbA1c), and proportion of patients with HbA1c < 6.5% (47.5 mmol/mL). From the “drug naïve” group of the study (patients with newly diagnosed T2DM), after matching for age, sex, BMI, drug dose and whether hypoglycemia occurred, 32 patients were selected and divided into 4 groups (with/without hypoglycemia treated with Xiaoke Pill or Glibenclamide, n = 8 for each group). Blood samples were drawn from the study participants at randomization and after 3 months of treatment.

### Treatment procedure

After a 4-week run-in period, eligible patients were randomized to receive double blind and double dummy therapy with oral Xiaoke or a Glibenclamide tablet. The starting dose for Glibenclamide was 1.25 mg/day, and the dose for Xiaoke was 5 pills per day (containing 0.25 mg Glibenclamide per pill, herb components including *Radix Puerariae*, *Radix Rehmanniae*, *Radix Astragali*, *Radix Trichosanthis*, *Stylus Zeae Maydis*, *Fructus Schisandrae Sphenantherae*, and *Rhizoma Dioscoreae*). The drug doses were adjusted every 4 weeks according to fasting serum glucose (FPG) levels: if FPG levels fell between 4.4 and 7.0 mmol/L, no adjustments were made; if FPG levels were higher than 7.0 mmol/L, an additional 5 Xiaoke pills or 1.25 mg of Glibenclamide was given; if FPG levels were lower than 4.4 mmol/L, then the dose was reduced. The maximum daily dose was 30 Xiaoke pills or 7.5 mg Glibenclamide.

### Ethics statement

The protocol for collecting the patient blood was approved by the Ethics Review Committee affiliated with each study center, and was implemented in accordance with provisions of the Declaration of Helsinki and Good Clinical Practice guidelines (*Trial Registration*: Chinese Clinical Trial Register Number, ChiCTR-TRC-08000074). All the participants provided their written informed consent to participate in this study.

### Tryptic digestion of the serum proteins

The ProteoMiner kit (Bio-Rad) with the hexapeptide ligand library was employed to reduce the high-abundance proteins influence according to the manufacturer’s instruction. Approximately 500 µg serum proteins were reduced using DTT followed by alkylation with IAM. The reduced protein was precipitated using pre-cooled acetone and remained at −20 °C for 6 h. The pellet was fully resolved in 0.5 M TEAB, pH 8.5 helped with sonication. In each group, 100 µg protein was digested using sequencing-grade trypsin at the ratio 1:20 (w/w, enzyme/protein) for 16 h at 37 °C. The protein concentration was determined using the Bradford method.

### Labeling the digested peptides with iTRAQ

An equal quantity of digested peptide in each group was reacted with one isobaric tag from the iTRAQ reagent (Applied Biosystems) in 70% isopropanol for 2 h at room temperature. In this study, the pooled sera were fully divided into 8 groups; thus, the iTRAQ isobaric tags with the m/z range 113–121 were used to label the different groups. After the labeling reactions were terminated, the 8 labeled groups were equally and well mixed for peptide separation and identification.

### LC for peptide separation and MS/MS for peptide identification

The peptides containing the mixed groups were diluted with buffer A, 10 mM KH_2_PO_4_ and 25% acetonitrile, pH 3, then loaded onto a strong cationic exchange (SCX) column (Phenomenex) with 25 cm × 4.6 mm (particle size at 5 µm, 100 A). The peptides were eluted using a gradient in which buffer B (10 mM KH_2_PO_4_ and 2 M KCL in 25% ACN, pH 3.0) and a total of 39 fractions were received for future experiments. The dried peptides were examined using UltraFlex MALDI TOF/TOF MS (Bruker) to estimate the peptide content in each eluted fraction. The fractionated peptides were further pooled into seventeen groups. Subsequently, the pooled peptides were loaded onto a reversed phase column, and the separated peptides were delivered into a TripleTOF 5600 MS instrument for further identification and quantification with parallel injections.

### Quantitative proteomics data analyses

The raw data (wiff) files acquired from the TripleTOF 5600 MS Q-TOF were converted into the Mascot Generic Format (mgf) using ProteinPilot (version 4.5.1). The MS/MS spectra were searched against the IPI-human database version 3.71 using the Mascot 2.3.3.01 software (Matrix Science). The data format files from the Mascot software were further analyzed using Scaffold software (version 2.0). The peptides were relatively quantified by dividing the reporter ion intensities between the reference and group. The protein differences were considered statistically significant at a *P* value <0.05. Cluster analyses were performed using R programming. The different proteins were functionally analyzed using IPA software online (http://pages.ingenuity.com/Ingenuity_Login.html).

### Multiple reaction monitoring (MRM) to verify the different proteins

Multiple reaction monitoring (MRM) was used to verify the different proteins determined using iTRAQ through the method reported by Zhang [11] with certain modifications. The tryptic peptides derived from serum were freeze-drying and reconstituting in 0.1% formic acid. Peptides were scanned by a QTRAP5500 mass spectrometer, and the MS/MS data was analyzed with Skyline to select the optimum peptides for MRM. The peptides followed the following criteria: (1) the peptides with unique sequence in the target protein; (2) a maximum m/z of peptide ≤1000 with a peptide length range 7–20 aa; (3) without C or M in peptides; and (4) no missed cleavage of trypsin. The MRM transitions for those selected peptides were listed for the MRM. Tryptic BSA as the internal standard was spiked into the serum peptides to normalize the loading amount.

## Results

### Results for the groups treated with the Xiaoke Pill or Glibenclamide

Of the 400 patients with new diagnosis of diabetes in the “Treatment Naive” group of the trial, 28 (15.2%) in the Xiaoke Pill group and 39 (21.2%) in the ‘Glibenclamide’ group experienced at least one episode of hypoglycemia [6]. We selected 8 patient sera pairs from each group (a pair from the sera before and after drug treatment) with a balanced distribution of gender, age and body mass index. Equal volumes of serum from 8 individual samples in each group were pooled for further proteomics analyses. The clinical biochemistry results for these patients were shown in Additional file 1: Table S1. A total of 8 pooled sera were used and denoted as follows: X_N_^B^, X_H_^B^, G_N_^B^, G_H_^B^, X_N_^A^, X_H_^A^, G_N_^A^ and G_H_^A^, in which X or G indicate treatment with the Xiaoke Pill or Glibenclamide, N or H indicate normal or hypoglycemic, and B or A indicate before or after the drug treatment.

### Experimental design for quantitative proteomics

**Fig. 1:**
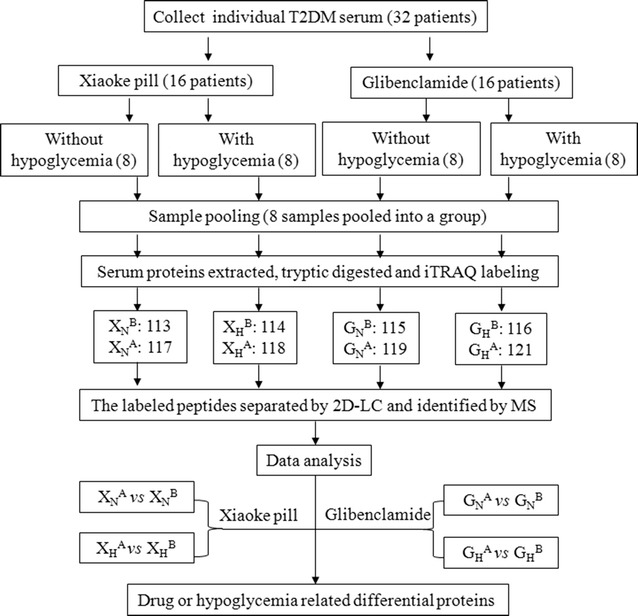
Scheme of the experimental design to survey the quantitative responses of serum proteins to the two drug treatments in T2DM. *X* Xiaoke Pill treatment, *G* Glibenclamide treatment, *B* before drug treatment, *A* after drug treatment; Number 113, 114, 115, 116, 117, 118, 119, 121: iTRAQ reporter

To detect the serum protein response to the Xiaoke Pill or Glibenclamide in T2DM patients, we used an 8-plex iTRAQ kit and labeled the digested peptides from the patient serum with the different individual tags. The experimental design is depicted in the flowchart (Fig. 1). Based on the serum sample groups, the iTRAQ tags were labeled as follows: 113 with X_N_^B^, 114 with X_H_^B^, 115 with G_N_^B^, 116 with G_H_^B^, 117 with X_N_^A^, 118 with X_H_^A^, 119 with G_N_^A^ and 121 with G_H_^A^, respectively. As mentioned above, the serum samples were grouped according to the following three conditions: with/without drug treatment (A/B), with/without hypoglycemia (H/N) and with/without the Xiaoke Pill (X/G); the serum without a drug treatment (B) was treated as the reference for relative quantification. Therefore, the changes in serum protein levels were quantitatively analyzed through two comparisons: the protein responses to the different drug treatments [i.e., X_N_^A^/X_N_^B^ (117/113) vs G_N_^A^/G_N_^B^ (119/115), X_H_^A^/X_H_^B^ (118/114) vs G_H_^A^/G_H_^B^ (121/116)] and the protein responses with/without hypoglycemia [i.e., X_H_^B^/X_N_^B^ (114/113) vs G_H_^B^/G_N_^B^ (116/115), X_N_^A^/X_N_^B^ (117/113) vs X_H_^A^/X_H_^B^ (118/114) and G_N_^A^/G_N_^B^ (119/115) vs G_H_^A^/G_H_^B^ (121/116)].

### Quality control of the quantitative data

**Fig. 2:**
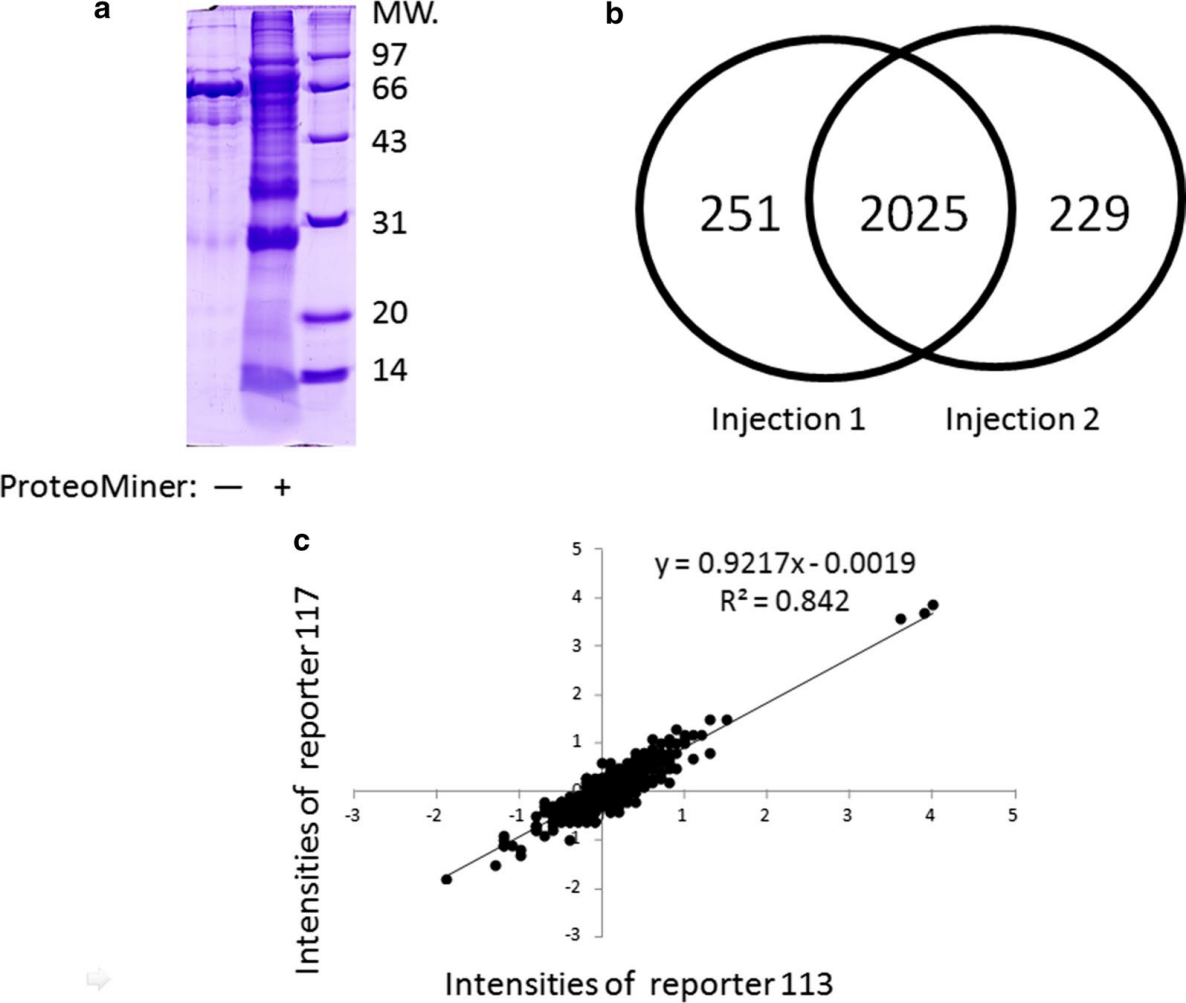
Quality control of proteomics analysis data. **a** *Left panel* SDS-PAGE for the serum without treatment by ProteoMiner; *Right panel* SDS-PAGE for the serum treated with ProteoMiner. **b** Reproducibility of the proteins identified in MS/MS duplicates. **c** The quantitative correlation analysis for the tag intensities 117 versus 113 (X_N_^A^/X_N_^B^)

To exclude the influence of high-abundance serum proteins in the proteomics analysis, ProteoMiner was used to compress the dynamic range of the serum proteins, and the effects of high abundance serum proteins compression were evaluated using SDS-PAGE (Fig. 2a). For an identified serum protein, we used stringent criteria and two unique peptides for a protein with a false discovery rate (FDR) of <5%. A total of 236 proteins were identified in the diabetes blood samples (Additional file 2: Table S2).

The reproducibility of the MS/MS signals was examined through duplicate injections. The identified peptides were verified with a >99% labeling efficiency. A total of 2276 and 2254 unique peptides with tags were identified through the parallel injections, and the overlap of unique peptides between two runs was approximately 89% as shown in Fig. 2b. Further, the MS/MS signals were evaluated and their quantitative information was discerned using tags and replicates. Figure 2c shows a typical comparison of the quantitative data, wherein the relative ratios of the tag intensities for 117 versus 113 (X_N_^A^/X_N_^B^) in one injection were plotted against the intensities from the other injection. Pearson correlation was used to estimate the correlation among the quantitative data; the linear regression slope was 0.84.

### Cluster analyses of the serum proteins identified among the patient groups

**Fig. 3:**
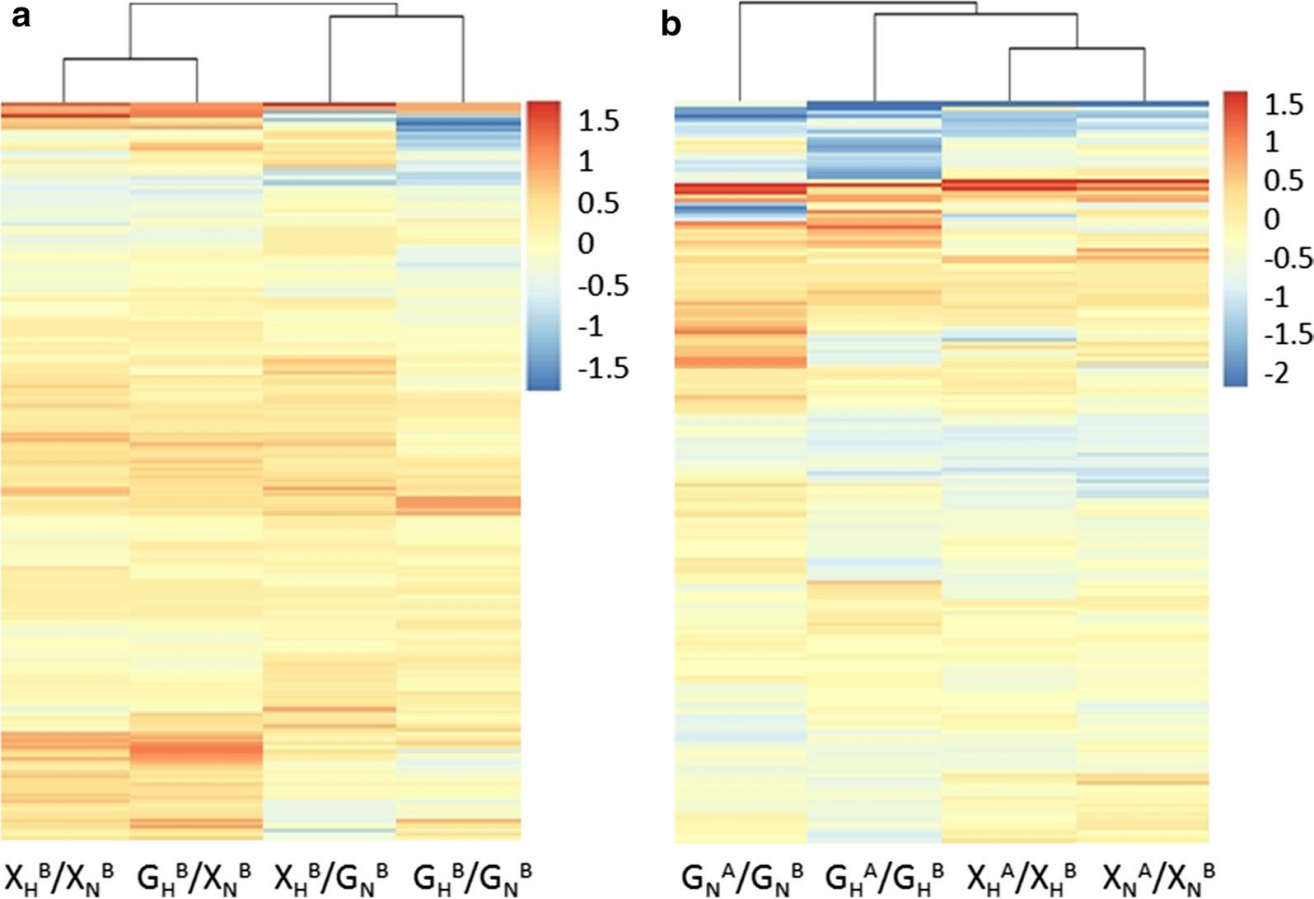
Cluster analysis of the abundance changes in the diabetic patient serum proteome in response to the Xiaoke Pill or Glibenclamide. **a** Cluster analysis of the abundance changes in serum proteins in response to T2DM with/without hypoglycemia without a drug treatment. **b** Cluster analysis of the abundance changes in serum proteins in response to the two different drug treatments

The distribution patterns of the serum protein abundance changes in response to hypoglycemia and the drug treatments were determined using cluster analyses through R programming. For the groups without a drug treatment, the two serum proteomes for the patients with and without hypoglycemia are comparable; the protein abundance ratios for X_H_^B^/X_N_^B^, G_H_^B^/X_N_^B^, X_H_^B^/G_N_^B^ and G_H_^B^/G_N_^B^ were expected to exhibit a similar pattern; otherwise, the ratios may indicate proteomic differences between the groups with and without hypoglycemia. Figure 3a displayed that the four abundance ratios were clustered to two patterns, X_H_^B^/X_N_^B^ and G_H_^B^/X_N_^B^ versus X_H_^B^/G_N_^B^ and G_H_^B^/G_N_^B^, implying that the X_H_^B^ and G_H_^B^ ratios were distributed similarly regardless of which group (X_N_^B^ or G_N_^B^) was referenced. Thus, we deduce that, without a drug treatment, the serum protein abundance in the two hypoglycemia groups (X_H_^B^ and G_H_^B^) should be similar but slightly different from the two diabetes groups without hypoglycemia. Next, we investigated whether the serum protein abundance was sensitive to the drug treatments. In calculating the abundance ratios, the serum abundance from diabetics without a drug treatment was used as the reference, and the four ratios, including X_N_^A^/X_N_^B^, X_H_^A^/X_H_^B^, G_N_^A^/G_N_^B^ and G_H_^A^/G_H_^B^ were generated. Figure 3b shows that the abundance changes of the serum proteins in X_N_^A^/X_N_^B^, X_H_^A^/X_H_^B^ and G_H_^A^/G_H_^B^ clustered into one group, wherein the two groups X_N_^A^/X_N_^B^ and X_H_^A^/X_H_^B^ exhibited a more comparable protein abundance distribution. G_N_^A^/G_N_^B^ exhibited a distribution pattern that differed from the other three patterns.

### The protein differences related to hypoglycemia and drug treatment

For an accurate quantitative comparison among the serum samples, we used two prerequisites to determine the significant changed proteins in serum: (1) the proteins used for quantitative comparison contained at least two unique peptides and significantly detectable tag signals and (2) a differential protein was defined with a fold change >1.3 between the two groups and with a *P* values <0.05, according to the variances of technical replicates to achieve 95% confidence.

As indicated in a cluster analysis of the serum samples without a drug treatment, we further investigated whether any protein differed significantly between the groups with and without hypoglycemia. The tag intensities of the T2DM serum proteins without hypoglycemia, such as X_N_^B^ and G_N_^B^, were used as references, and the serum protein abundance differences between the groups without and with hypoglycemia were estimated using the Scaffold algorithm. The quantitative analyses revealed that only one protein, C-reactive protein, was up-regulated in hypoglycemia groups.

Which serum proteins exhibited changes in abundance due to drug treatment in the T2DM patients? The T2DM serum protein tag intensities before the drug treatment, such as in X_N_^B^, X_H_^B^, G_N_^B^ and G_H_^B^, were used as the references, and the abundance differences between the serum proteins before and after the drug treatment were estimated using the Scaffold algorithm (Additional file 3: Table S3). Twenty-five and 21 proteins exhibited different abundances between the with/without Xiaoke Pill treatment in the without hypoglycemia group and the hypoglycemia group, respectively; 24 and 25 proteins exhibited different abundances between the groups with and without the Glibenclamide treatment in the without hypoglycemia group and the hypoglycemia group, respectively.

In the “no hypoglycemia” groups, 5 proteins, including inter-alpha-trypsin inhibitor heavy chain H4, cholesteryl ester transfer protein, apolipoprotein C, histone H2B and alpha-2-macroglobulin, exhibited the same abundance responses to both drugs. In the with hypoglycemia groups, 8 proteins, including histone H2B, apolipoprotein C, alpha-2-macroglobulin, inter-alpha-trypsin inhibitor heavy chain H1, inter-alpha-trypsin inhibitor heavy chain H4, complement component C9, biotinidase and complement component C8 gamma chain, exhibited the same abundance changes among the two groups.

**Fig. 4:**
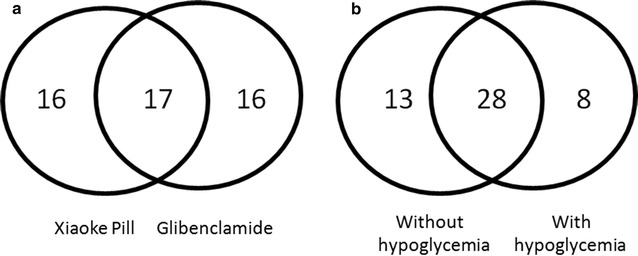
Venn diagram of the serum proteins with different abundances related to a drug treatment or hypoglycemia in T2DM patients. **a** Venn diagram of the proteins with different abundances related to the Xiaoke Pill or Glibenclamide treatment. **b** Venn diagram of the proteins with different abundances related to hypoglycemia

In total, 49 proteins exhibited abundance differences after an anti-diabetic drug treatment, including 33 that responded to the Xiaoke Pill and 33 that responded to Glibenclamide, and 17 proteins overlapped between the two drug treatments (Fig. 4a). Considering the total proteins that exhibited abundance differences are grouped based on the sera with or without hypoglycemia, 41 proteins belong to the T2DM without hypoglycemia group, and 36 belong to the hypoglycemia group. Twenty-eight proteins that exhibited abundance differences overlapped between the sera with and without hypoglycemia (Fig. 4b). The percentage of overlapping proteins that exhibited abundance differences after the two drug treatments (34%) is remarkably lower than among the two serum groups with/without hypoglycemia (57%).

### Verification of the proteins with different abundances by MRM

Based on our principles for selecting the peptides and transitions for MRM, among the 49 candidates obtained from the iTRAQ analyses, 32 proteins with abundance differences were selected for MRM validation. Through a quantitative analysis using Skyline 1.4, approximately 68% of the quantitative results from MRM were consistent with the iTRAQ results (Additional file 3: Table S3).

## Discussion

Quantitative proteomics has been widely employed in serum proteomics, especially for labeling and quantification, to discover disease-related protein biomarkers or examine serum protein responses to medical perturbation. For instance, Yanagida et al. [12] used iTRAQ to label peptides from serum proteins collected from rheumatoid arthritis (RA) patient and discern serum protein changes in response to a tocilizumab treatment. Using a combination strategy, Saminathan et al. [13] gathered sera from the patients with coagulopathy, and quantitatively evaluated the serum protein changes after a warfarin treatment, which is a commonly prescribed oral anticoagulant. We realized that the quantitative proteomics technical challenges for the serum proteome differ from a similar analysis using cells or tissue lysates. First, the highly abundant proteins are always potential interferences for quantitating serum proteins. Second, the dynamic variations among the individual serum samples are relative large; thus, the protein signals of interest are not easily distinguished from the varied sera backgrounds. Thus, it is necessary to modify the traditional iTRAQ strategy for serum proteomics. In this project, we adopted three optimization steps: (1) collect the pooled serum samples that are expected avoidable in large variations of serum protein abundance, (2) employ a cluster analysis to provide a global perspective on the protein abundance similarities among the groups, and (3) define the proteins that respond differently to the drug treatment based on a stringent criterion. With this stepwise analysis, we provide convincing evidence that likely closely reflects the serum protein responses to the Xiaoke Pill or Glibenclamide in T2DM patients.

In our early observations, we found that clinical application of the Xiaoke Pill to T2DM patients significantly reduced the risk of hypoglycemia induced by Glibenclamide [6]. The pharmaceutical mechanism for this improvement has not been determined. As serum is a major carrier for oral drugs and a sensor responsible for disseminating the changing proteins throughout the body, we reason that the qualitative and quantitative serum protein changes may contain information on the serum or tissue protein responses to drug treatments. Therefore, quantitative proteomics using iTRAQ is expected to provide at least two sets of information regarding treatment with the Xiaoke Pill and Glibenclamide. First, is the serum protein abundance in the T2DM patients sensitive to the anti-diabetic drugs? If so, do these changes correspond to the two different drugs in a similar manner? Furthermore, which serum proteins can be defined as the significant responders to a drug? Next, does the serum protein abundance correlate with hypoglycemia? Is there a relationship between the serum protein abundance responses to the drugs and hypoglycemia? As demonstrated by the results in Fig. 3, the protein abundance changes in response to the Xiaoke Pill treatment regardless of whether the group exhibited hypoglycemia differed from the Glibenclamide treatment. Further, we analyzed the proteomes for abundance differences, wherein the protein that exhibited differences induced by the Xiaoke Pill clearly differed from the Glibenclamide-induced changes; <34% of the proteins overlapped. Hence, the quantitative proteomics experiment herein clearly shows that the two anti-diabetic drugs, the Xiaoke Pill and Glibenclamide, perturb the abundance distributions of serum proteins in a distinctive manner. The proteins that exhibit difference related to the drug treatment are likely candidates for further studies on the underlying pharmaceutical mechanism and biomarker validation, especially for the Xiaoke Pill. We considered whether the serum protein abundance correlates with hypoglycemia status. Without a drug treatment, the abundance change in the serum proteins was only slight between with and without hypoglycemia groups; however, in further analyses of these proteins, only one was identified as responsive to hypoglycemia. The current quantitative proteomics data support the notion that a specific abundance distribution or biomarker of serum proteins indicative of hypoglycemia does not exist. Moreover, considering the early clinical application evidence for the two drugs and the different sets of proteins that exhibit abundance changes related to the two drugs, we deduce that the serum protein abundance response to the anti-diabetic drugs might be associated with hypoglycemia symptoms. Regarding the specific protein(s) involved in hypoglycemia, further mechanism studies are necessary.

Notably, certain serum proteins were identified as dependent on anti-diabetic drugs for the first time, including angiotensinogen, alpha-1-antitrypsin (AAT), paraoxonase (PON1) and fibulin. The renin-angiotensin system (RAS) is well known for its key role in regulation of vasoconstriction and fluid homeostasis. Recently, it was found that some major components of RAS expressed in various tissues including adipose tissue and the blockade of RAS could improve insulin sensitivity [14–16]. Moreover, as one of the pro-inflammatory adipokines, overproduction of angiotensinogen from adipose tissue was found to induce adipose inflammation, glucose intolerance and insulin resistance [17]. In adipose-specific angiotensinogen knockout mice (Agt-KO), reduced adipose inflammation and increased glucose tolerance were observed partly via increased metabolic activity of adipose cells [18]. Based on several large-scale clinical studies, Chu et al. [19] suggested that Ang II involves in regulating insulin secretion by the pancreatic beta-cell and insulin sensitivity in the peripheral tissue. These findings are consistent with the present study that angiotensinogen decreased in Xiaoke Pill treatment and without hypoglycemia group. AAT is a member of the serine protease inhibitor (serpin) superfamily, which has a key role in maintaining protease–antiprotease balance and is one of the major protective circulating proteins in humans. It has been reported that the level of AAT increased and the capacity of serum trypsin inhibitory decreased in diabetic patients [20–22]. There is reason to speculate that deficiency of AAT may be associated with an increased risk of developing T2DM and the abnormal response to anti-diabetic drugs. Serum PON1 is synthesized in the liver and is an HDL-associated enzyme that hydrolyzes organophosphate compounds and fatty acid lactones. Several previous reports have documented that PON1 gene polymorphisms and lower PON1 activity were linked with the risk of T2DM and diabetic complications [23–25]. Rosenblat et al. [26] recently demonstrated that PON1 plays a protective role against diabetes development, and the high concentrations of glucose in diabetic patient may account for PON1 dissociation from HDL. However, relatively little information is available on the relationship between circulating PON1 mass and diabetic therapy. In the present analysis, we demonstrated that the activity of PON1 may serve as a novel marker to guide the selection of anti-diabetic drugs. Fibulin-1 is an elastin-associated matrix molecule that is found in elastic fibers and basement membranes in blood vessels. Scholze et al. [27] reported that increased plasma fibulin-1 levels were associated with diabetes and impaired kidney function. In a longitudinal cohort study, higher concentrations of fibulin-1-protein were detected in artery extracts from patients with diabetes than individuals without diabetes, and the concentrations of plasma fibulin-1 were correlated with glucose control [28]. Our observation of the changes of fibulin-1 before and after anti-diabetic treatment may provide clues to explain why some anti-diabetic drugs have additional cardiovascular benefits.

How the anti-diabetic drugs regulate the diabetes-specific serum proteins is an attractive topic for understanding the pharmaceutical mechanisms. This work is only a view from the proteomics changes and it will provide more information about the drug action if the lipidomics and metabolomics study were combined. In addition, the peptidome and proteome in urine also could provide useful clues to illustrate the effects of drugs against the diabetes.

## Conclusions

We employed the quantitative proteomics and statistics approach to evaluate the proteomic abundance changes in the T2DM sera after treatment of the anti-diabetic drugs, Xiaoke Pill and Glibenclamide. We observed that the two T2DM drugs indeed perturbed the serum proteome abundance in the diabetic patients, whereas the quantitative profiles of serum proteome in the T2DM with/without hypoglycemia were basically comparable. This study lays the background for new line of research to develop serum protein markers based prediction paradigm for the treatment of patients with T2DM.

## Additional files

**Additional file 1: Table S1.** Clinical characteristic of biochemistry for the T2DM patients.

**Additional file 2: Table S2.** The list of 236 identified serum proteins in iTRAQ.

**Additional file 3: Table S3.** The serum proteins with different abundances in T2DM in response to the anti-diabetic drugs.

## Abbreviations

T2DM: type 2 diabetes mellitus
HbA1c: glycated hemoglobin
TCM: traditional Chinese medicine
iTRAQ: isobaric tags for relative and absolute quantitation
MRM: multiple reaction monitoring
FDR: false discovery rate
AAT: alpha-1-antitrypsin
PON1: paraoxonase
Ang II: angiotensin II
HDL: high density lipoprotein
