# Supplementary material for: The serum protein responses to treatment with Xiaoke Pill and Glibenclamide in type 2 diabetes patients
Source: Clin Proteomics. 2017 May 17;14:19. doi: 10.1186/s12014-017-9154-0 (PMC5436452; doi:10.1186/s12014-017-9154-0)
Supplement: Supplementary file 1 — Additional file 1: Table S1. Clinical characteristic of biochemistry for the T2DM patients. [file 12014_2017_9154_MOESM1_ESM.docx]

**Supplementary Table**

Supplementary Table 1. Clinical characteristic of biochemistry for the T2DM patients

| Variable | Xiaoke Pill Group | | Glibenclamide Group | | |
| --- | --- | --- | --- | --- | --- |
|  |  |  |  |  |  |
|  | without hypoglycaemia | with hypoglycaemia | | without hypoglycaemia | with hypoglycaemiae |
| Age (years) | 56±6 | 56±8 | | 57±8 | 59±3 |
| BMI (kg/m^2^) | 24.6±2.2 | 25.1±1.7 | | 25.3±1.5 | 23.9±2.4 |
| SBP (mmHg) | 136±15 | 133±17 | | 121±12 | 132±24 |
| DBP (mmHg) | 84±8 | 76±9 | | 84±9 | 81±9 |
| HbA1c (%) | 8.3±1.1 | 8.6±1.1 | | 8.1±1.1 | 7.7±0.4 |
| FPG (mmol/L) | 9.5±1.4 | 9.5±1.8 | | 8.5±1.9 | 8.7±1.0 |
| FINS (mIU/L) | 14.2±11.3 | 10.7±10.3 | | 10.7±6.1 | 10.2±8.9 |
| TG (mmol/L) | 1.8±0.6 | 1.4±0.5 | | 1.7±0.4 | 1.8±0.6 |
| TC (mmol/L) | 4.6±1.0 | 4.9±1.0 | | 5.1±0.9 | 5.0±0.9 |
| HDL-C (mmol/L) | 1.1±0.2 | 1.3±0.2 | | 1.1±0.3 | 1.2±0.5 |
| LDL-C (mmol/L) | 3.1±0.9 | 3.3±1.1 | | 3.6±0.7 | 3.4±0.9 |

The data was recorded from 8 individuals, 4 female and 4 male, BMI means body mass index, DBP means diastolic blood pressure, FINS means fasting insulin, FPG means fasting plasma glucose, HbA1C means hemoglobin A1c, HDL-C means high-density lipoprotein cholesterol, LDL-C means low-density lipoprotein cholesterol, SBP means systolic blood pressure, TC means total cholesterol, TG means triglyceride.
