# Supplementary material for: The serum protein responses to treatment with Xiaoke Pill and Glibenclamide in type 2 diabetes patients
Source: Clin Proteomics. 2017 May 17;14:19. doi: 10.1186/s12014-017-9154-0 (PMC5436452; doi:10.1186/s12014-017-9154-0)
Supplement: Supplementary file 3 — Additional file 3: Table S3. The serum proteins with different abundances in T2DM in response to the anti-diabetic drugs. [file 12014_2017_9154_MOESM3_ESM.docx]

**Supplementary Table**

Supplementary Table 3. The serum proteins with different abundances in T2DM in response to the anti-diabetic drugs

| **Drug Treatment** | **Proteins** | **iTRAQ** | | **MRM** | | | **Match** |
| --- | --- | --- | --- | --- | --- | --- | --- |
|  |  | **Fold Change** | **+SD** | | **Fold Change** | **+SD** |  |
| Xiaoke Pill treatment without hypoglycemia | Histone H2B type 2-E | 0.36 | 0.17 | | - | - | - |
|  | Angiotensinogen | 0.48 | 0.22 | | 0.01 | 0.01 | Y |
|  | Histone H4 | 0.49 | 0.03 | | - | - | - |
|  | Isoform 1 of L-lactate dehydrogenase A chain | 0.62 | 0.10 | | - | - | - |
|  | Apolipoprotein A-IV | 0.64 | 0.05 | | 0.39 | 0.04 | Y |
|  | Isoform 1 of C-reactive protein | 0.65 | 0.14 | | - | - | - |
|  | Isoform 1 of inter-alpha-trypsin inhibitor heavy chain H4 | 0.71 | 0.16 | | 0.41 | 0.02 | Y |
|  | Serum amyloid A protein | 0.72 | 0.08 | | 0.60 | 0.08 | Y |
|  | Serum paraoxonase/lactonase 3 | 0.73 | 0.02 | | 0.41 | 0.08 | Y |
|  | Chromogranin-A | 0.73 | 0.09 | | - | - | - |
|  | Isoform 1 of alpha-1-antitrypsin | 0.76 | 0.17 | | 0.09 | 0.01 | Y |
|  | Fibulin-5 | 0.77 | 0.02 | | - | - | - |
|  | Apolipoprotein C-III | 0.78 | 0.09 | | 0.19 | 0.03 | Y |
|  | Isoform 1 of pregnancy zone protein | 0.78 | 0.05 | | - | - | - |
|  | Isoform 1 of complement factor H | 1.41 | 0.09 | | 0.49 | 0.08 | N |
|  | Complement C5 | 1.43 | 0.13 | | 0.64 | 0.19 | N |
|  | Alpha-2-macroglobulin | 1.43 | 0.08 | | 0.22 | 0.06 | N |
|  | Complement component 1, q subcomponent, B chain precursor | 1.53 | 0.08 | | 0.56 | 0.10 | N |
|  | Prolow-density lipoprotein receptor-related protein 1 | 1.55 | 0.02 | | - | - | - |
|  | Complement C1q subcomponent subunit A | 1.55 | 0.04 | | 1.54 | 0.15 | Y |
|  | Complement C1q subcomponent subunit C | 1.57 | 0.08 | | 0.61 | 0.10 | N |
|  | Isoform 1 of cholesteryl ester transfer protein | 1.63 | 0.16 | | - | - | - |
|  | Keratin, type II cytoskeletal 2 epidermal | 1.85 | 0.05 | | - | - | - |
|  | Keratin, type II cytoskeletal 1 | 1.87 | 0.13 | | 0.21 | 0.15 | N |
|  | Inter-alpha (Globulin) inhibitor H2, isoform CRA_a | 2.04 | 0.26 | | 2.60 | 0.18 | Y |
| Xiaoke Pill treatment with hypoglycemia | Isoform 1 of ficolin-2 | 0.31 | 0.10 | | 0.40 | 0.04 | Y |
|  | Isoform 1 of L-lactate dehydrogenase A chain | 0.56 | 0.07 | | - | - | - |
|  | Isoform 1 of inter-alpha-trypsin inhibitor heavy chain H4 | 0.57 | 0.11 | | 0.61 | 0.10 | Y |
|  | Isoform 1 of C-reactive protein | 0.58 | 0.14 | | - | - | - |
|  | Histone H2B type 2-E | 0.61 | 0.05 | | - | - | - |
|  | Serum amyloid A protein | 0.61 | 0.13 | | 0.60 | 0.17 |  |
|  | Complement component C9 | 0.63 | 0.13 | | 0.36 | 0.07 |  |
|  | Complement component 6 precursor | 0.66 | 0.04 | | 0.40 | 0.05 | Y |
|  | Fibulin-5 | 0.69 | 0.05 | | - | - | - |
|  | Complement component C8 gamma chain | 0.71 | 0.08 | | - | - | - |
|  | Prolow-density lipoprotein receptor-related protein 1 | 0.71 | 0.02 | | - | - | - |
|  | Complement component C8 beta chain | 0.73 | 0.13 | | 0.36 | 0.07 | Y |
|  | Isoform 1 of Alpha-1-antitrypsin | 0.74 | 0.12 | | 0.17 | 0.03 |  |
|  | Apolipoprotein C-III | 0.74 | 0.11 | | 0.42 | 0.08 |  |
|  | Complement C5 | 0.76 | 0.10 | | - | - | - |
|  | Inter-alpha-trypsin inhibitor heavy chain H1 | 0.77 | 0.17 | | 0.43 | 0.09 |  |
|  | Apolipoprotein A-IV | 0.78 | 0.08 | | 0.63 | 0.04 |  |
|  | Keratin, type I cytoskeletal 10 | 1.35 | 0.10 | | - | - | - |
|  | Alpha-2-macroglobulin | 1.51 | 0.09 | | 0.37 | 0.06 | N |
|  | Biotinidase | 1.53 | 0.06 | | 0.40 | 0.02 | N |
|  | Chromogranin-A | 2.62 | 0.18 | | - | - | - |
| Glibenclamide treatment without hypoglycemia | Keratin, type II cytoskeletal 1 | 0.28 | 0.03 | | 0.61 | 0.15 | Y |
|  | Keratin, type II cytoskeletal 2 epidermal | 0.35 | 0.08 | | - | - | - |
|  | Isoform 1 of ficolin-2 | 0.46 | 0.12 | | 0.67 | 0.09 | Y |
|  | Keratin, type I cytoskeletal 10 | 0.48 | 0.06 | | - | - | - |
|  | Histone H2B type 2-E | 0.54 | 0.14 | | - | - | - |
|  | Haptoglobin | 0.57 | 0.13 | | 1.59 | 0.32 | N |
|  | Isoform 1 of inter-alpha-trypsin inhibitor heavy chain H4 | 0.65 | 0.11 | | 0.27 | 0.02 | Y |
|  | Complement component C7 | 0.66 | 0.12 | | 0.42 | 0.07 | Y |
|  | Isoform 1 of fibrinogen alpha chain | 0.69 | 0.09 | | - | - | - |
|  | Fibrinogen beta chain | 0.69 | 0.15 | | - | - | - |
|  | Complement component C9 | 0.71 | 0.12 | | 0.41 | 0.15 | Y |
|  | Isoform 1 of serum albumin | 0.73 | 0.13 | | 0.20 | 0.05 | Y |
|  | Complement component C8 gamma chain | 0.73 | 0.14 | | - | - | - |
|  | Isoform gamma-B of fibrinogen gamma chain | 0.73 | 0.16 | | - | - | - |
|  | Inter-alpha-trypsin inhibitor heavy chain H1 | 0.74 | 0.14 | | 0.26 | 0.03 | Y |
|  | Protein AMBP | 0.75 | 0.22 | | 0.41 | 0.05 | Y |
|  | Complement component 6 precursor | 0.75 | 0.13 | | 0.42 | 0.06 | Y |
|  | Apolipoprotein C-III | 0.76 | 0.11 | | 0.21 | 0.06 | Y |
|  | Isoform LMW of kininogen-1 | 0.77 | 0.05 | | 0.18 | 0.10 | Y |
|  | Alpha-2-macroglobulin | 1.32 | 0.10 | | 0.35 | 0.05 | N |
|  | Serum paraoxonase/arylesterase 1 | 1.41 | 0.13 | | 0.89 | 0.16 | N |
|  | Angiotensinogen | 1.62 | 0.17 | | 1.91 | 0.28 | Y |
|  | Isoform 1 of cholesteryl ester transfer protein | 1.74 | 0.25 | | - | - | - |
|  | Biotinidase | 1.87 | 0.14 | | 0.66 | 0.17 | N |
| Glibenclamide treatment with hypoglycemia | Inter-alpha-trypsin inhibitor heavy chain H1 | 0.57 | 0.06 | | 0.64 | 0.08 | Y |
|  | Protein AMBP | 0.62 | 0.10 | | 0.93 | 0.19 | N |
|  | Histone H2B type 2-E | 0.63 | 0.08 | | - | - | - |
|  | Apolipoprotein C-III | 0.69 | 0.07 | | 0.22 | 0.04 | Y |
|  | Complement component C9 | 0.71 | 0.03 | | - | - | - |
|  | Isoform LMW of kininogen-1 | 0.72 | 0.04 | | 0.54 | 0.10 | Y |
|  | Complement component C8 gamma chain | 0.75 | 0.09 | | - | - | - |
|  | Isoform 1 of inter-alpha-trypsin inhibitor heavy chain H4 | 0.76 | 0.03 | | 0.38 | 0.05 | Y |
|  | A30 (fragment) | 0.76 | 0.10 | | - | - | - |
|  | Complement C5 | 1.31 | 0.15 | | 0.93 | 0.16 | N |
|  | Biotinidase | 1.32 | 0.13 | | 1.39 | 0.05 | Y |
|  | Alpha-2-macroglobulin | 1.32 | 0.08 | | 1.84 | 0.06 | Y |
|  | Isoform 1 of phosphatidylinositol-glycan-specific phospholipase D | 1.34 | 0.16 | | 1.64 | 0.04 | Y |
|  | Beta-Ala-His dipeptidase | 1.41 | 0.28 | | 1.70 | 0.25 | Y |
|  | Ceruloplasmin | 1.72 | 0.13 | | 3.12 | 0.31 | Y |
|  | Keratin, type II cytoskeletal 2 epidermal | 1.74 | 0.17 | | 0.42 | 0.07 | N |
|  | Serum paraoxonase/arylesterase 1 | 2.03 | 0.12 | | 1.90 | 0.26 | Y |
|  | Pleckstrin | 2.06 | 0.03 | | - | - | - |
|  | Angiotensinogen | 2.12 | 0.08 | | 1.55 | 0.12 | Y |
|  | Alpha-1-antichymotrypsin | 2.14 | 0.14 | | 0.70 | 0.17 | N |
|  | Monocyte differentiation antigen CD14 | 2.83 | 0.12 | | - | - | - |
|  | Isoform 1 of alpha-1-antitrypsin | 3.73 | 0.13 | | 1.33 | 0.16 | N |
|  | Isoform 1 of fibrinogen alpha chain | 12.21 | 0.27 | | - | - | - |
|  | Fibrinogen beta chain | 13.83 | 0.31 | | - | - | - |
|  | Isoform gamma-B of fibrinogen gamma chain | 14.73 | 0.14 | | - | - | - |

Note: "-"represents without qualifying peptides and transitions for the MRM experiment.
